# Citation analysis of the 100 top-cited articles on discectomy via endoscopy since 2011 using alluvial diagrams: bibliometric analysis

**DOI:** 10.1186/s40001-022-00782-0

**Published:** 2022-09-01

**Authors:** Chao-Hung Yeh, Tsair-Wei Chien, Po-Hsin Chou

**Affiliations:** 1 Department of Neurosurgery, Chi Mei Medical Center, Tainan 700, Taiwan; 2 Department of Optometry, Chung Hwa University of Medical Technology, Tainan, Taiwan; 3 Medical Research Department, Chi-Mei Medical Center, Tainan, Taiwan; 4 Department of Orthopedics and Traumatology, Taipei Veterans General Hospital, Taipei, Taiwan; 5 School of Medicine, National Yang Ming Chiao Tung University, Taipei, Taiwan

**Keywords:** Bibliometric, Citation analysis, Medical subject heading, Percutaneous endoscopic interlaminar discectomy, Alluvial diagram, Web of Science, PubMed, Social network analysis

## Abstract

**Background:**

Percutaneous endoscopic lumbar discectomy (PELD) is synonymous with percutaneous endoscopic transforaminal discectomy (PETD) and percutaneous endoscopic interlaminar discectomy (PEID). PEID has gained increasing recognition for its small incision, quick recovery, short hospital stay, and equivalent clinical outcome to open surgery. Numerous articles related to PEID have been published in the literature. However, which countries, journals, subject categories, and articles have ultimate influence remains unknown. The study aimed to (1) display influential entities in 100 top-cited PEID-related articles (T100PEID) on the alluvial diagram and (2) investigate whether medical subject headings (i.e., MeSH terms) can be used to predict article citations.

**Methods:**

T100PEID data can be found since 2011 in the PubMed and Web of Science (WOS) databases. Using alluvial diagrams, citation analysis was conducted to compare the dominant entities. We used social network analysis (SNA) to classify MeSH terms and research areas extracted from PubMed and WOS. The difference in article citations across subject categories and the predictive power of MeSH terms on article citations in T100 PEID were examined using one-way analysis of variance (ANOVA) and regression analysis.

**Results:**

A total of 81% of T100PEID is occupied by the top three countries (the US, China, and South Korea). There was an overall T100PEID impact factor of 41.3 (IF = citations/100). Articles were published in *Spine (Phila Pa 1976)* (23%; IF = 41.3). Six subject categories were classified using the SNA. The most cited article authored by D Scott Kreiner from Ahwatukee Sports and Spine in the US state of Phoenix had 123 citations in PubMed. The network characteristics of T100PEID are displayed on the alluvial diagram. No difference was found in article citations among subject categories (*F* = 0.813, *p* = 0.543). The most frequently occurring MeSH term was surgery. MeSH terms were evident in the prediction power of the number of article citations (*F* = 15.21; *p* < 0 .001).

**Conclusion:**

We achieved a breakthrough by displaying the T100PEID network characteristics on the alluvial plateau. The MeSH terms can be used to classify article subject categories and predict T100PEID citations. The alluvial diagram can be applied to bibliometrics on 100 top-cited articles in future studies.

**Supplementary Information:**

The online version contains supplementary material available at 10.1186/s40001-022-00782-0.

## Keypoints

- An Alluvial diagram was drawn to display the network characteristics of T100PEID, which is novel and modern in the literature.
- The method of drawing the Alluvial demonstrated in detail with documents in supplemental digital contents can be applied to make bibliometric studies brief, concise, and powerful.
- The impact beam plot (IBP) is an additional visualization introduced in this study. The online IBP was demonstrated and worthy of future similar studies to highlight the most influential articles with a glance at a picture.

**Supplementary Information:**

The online version contains supplementary material available at 10.1186/s40001-022-00782-0.

## Background

The percutaneous endoscopic lumbar discectomy (PELD) procedure has significantly evolved over the past decade since Kambin [1] developed and applied an arthroscopic technique to treat lumbar disc herniation (LDH). PELD refers to percutaneous endoscopic transforaminal discectomy (PETD) [2.3] and percutaneous endoscopic interlaminar discectomy (PEID) [4] as minimally invasive spinal procedures, which have gained increasing recognition for their small incision, rapid recovery, brief hospital stay, and equivalent clinical outcomes to open surgery [5]. PETD and PEID are comparable to open spine surgery or other minimally invasive surgeries in efficacy and safety [6]. The prevalence of PELD has led to an increase in surgery-related complications [7, 8]. To obtain satisfactory clinical efficacy, sufficient consideration should be given to the indication of PELD (5).

### PEID as a minimally invasive spinal procedure

As a significant complement to PETD, PEID is also suitable for highly migrated or calcified disc herniation because of the large open space in the spinal canal [9–11]. However, PEID requires traction of the thecal sac to deal with disc fragments, which may consequently cause dural laceration and other complications [5].

Using the PubMed database and keywords ((((Endoscopic discectomy [MeSH Terms]) OR (Endoscopic discectomy[MeSH Terms])) OR (Percutaneous endoscopic transforaminal discectomy or discectomy [MeSH Terms])) OR (Percutaneous endoscopic interlaminar discectomy or diskectomy[MeSH Terms])), over 6,670 articles were found. We were motivated to explore the article features of PEID by collecting 100 top-cited articles (T100PEID for short) and then reporting influential entities (e.g., authors, institutes, countries, subject categories, document types, and themes) based on article citations.

### Bibliometric analysis and graphical study

Bibliometrics refers to a form of statistical analysis that has been widely used to analyze published articles, offering an efficient way to measure the scientific impact of articles by employing mathematical models and techniques in a particular field [13, 14]. In this type of analysis, entities (e.g., countries, institutions, and authors) with the most significant scientific contributions are identified. Citation counts typically indicate researchers’ interest in using journal articles in their research [17]. Hence, bibliometric analyses can identify study hotspots and future trends in a particular field of study [18, 19].

In PubMed [20], a search of titles containing the phrase “100 top-cited” retrieved 198 publications. There were three categories of information in these articles: (i) descriptive statistics (DS), (ii) significant topics or article types with research domains (RD), and (iii) research achievements in entities (RA) [21]. By using citation analysis, the most influential articles were identified in orthopedic surgery [22], subspecialties of arthroscopy [23, 23], foot and ankle [24, 24], arthroplasty [25], and pediatric orthopedics [26], and spinal deformity [27–29].

Although those studies helped us identify the main features that created an enormous distinction within the field and provided a guideline for physicians and researchers in a discipline, two perspectives were frequently ignored owing to the lack of (1) a visualization for highlighting all relevant entities on a picture [30–32] and (2) a way to predict the number of article citations for the future [33–36].

### Study aims

Our research goals were to (1) display influential entities in T100PEID on the alluvial diagram [37, 38] and (2) investigate whether medical subject headings (i.e., MeSH terms) could be used to predict article citations.

## Methods

### Data source

A two-step process was used to arrange the data. Initially, the authors searched the PubMed database using the keywords (“:2011”[Date—Publication]: “3000”[Date—Publication]) and ((((Endoscopic diskectomy[MeSH Terms]) OR (Endoscopic discectomy[MeSH Terms])) OR (Percutaneous endoscopic transforaminal discectomy or diskectomy[MeSH Terms])) as of April 12, 2022, and matched articles to the number of citations in PubMed and Web of Science (WOS). The relevant metadata (e.g., years, countries of origin, research categories, document types, publishing journals, medical subject heading, MeSH terms) were collected from T100PEID [39].

Second, based on the article metadata, two parts were included: (1) visualizations using alluvial [37, 38] to display all relevant entities and their associations and (2) inferring statistics using MeSH terms to predict citations for articles in addition to DS, RD, and RA [21], as in traditional bibliographical studies [21–31].

This study does not require ethical approval, as all data were obtained from a publicly available database.

### Data arrangements and presentations

We extracted major entities from each article: title, abstract, authors, publication year, country of origin, citation count, journal, identity number in PubMed (PMID), and major topic MeSHs. If the authors had more than one affiliation from different countries, the first affiliation was used as the country of origin. Major topic MeSHs (with the symbol of asterisk for each article in PubMed) were involved, and subheadings were removed.

#### Descriptive statistics (DS)

Two tables were produced for presenting (1) the contributions denoted by publications and citations from countries/regions and journals over the years, respectively. The independence t-test was performed to examine article citation differences between WOS and PubMed.

#### Major topics or article types with research domains (RD)

Article subject categories (based on research areas in WOS and MeSH terms in PubMed) were clustered using social network analysis (SNA) [31] and Pajek software [40]. The closer terms appeared in an identical subnetwork. Relevant terms gathered in subject categories were highlighted on a dynamic visual board as the mode of the traditional word cloud [42]. The terms with the most frequency in the respective clusters represent the themes highlighted by SNA. One-way analysis of variance (ANOVA) was performed to examine the difference in article citations among subject categories.

#### Research achievements (RA)

Citation analysis was applied to understand the RAs that contributed to PEID among subject categories and significant MeSH terms using the pyramid plot [41].

The T100ADHDs [39] since 2011 were dotted on the impact beam plot (IBP) [43, 44] using the citation percentiles (i.e., with the MSExcel function of percentrank()) to display the article impact from 0 to 100 by year (based on normalized citations for each article). The overall hT-index [46.47] and h-index [15] were compared with the median score on the IBP using the online technique [47]. The red dots in IBP represent the clinical research compared to the counterparts denoted by black dots.

#### Visualizations using the alluvial diagram

One look is worth a thousand words and quite a few numbers [48]. The alluvial diagrams [37, 38] were drawn based on article numbers and hT indices in T100BEID for entities, including years, countries, institutes, document types, subject categories, and PMID. The more proportional publications (or citations) in the alluvial diagram would have more giant blocks in height and flow. The red-colored flows represent the selected entity directly associated with other entities on the alluvial diagram.

#### Inferring statistics using MeSH terms to predict article citations

The impact factors (Ifs) of MeSH terms were computed based on equal-size proportions and citations in an article [17]. The weighted scores yielded by MeSH weights (i.e., the number of citations per article) in each article were used to predict the original citations [17, 21, 49]. Regression analysis was applied to examine the prediction power of MeSH terms on article citations in T100 PEID.

### Statistics and tools

Visual representation on a dashboard was developed to present the research results. Author-made modules (1) made all Figures in Excel (Microsoft Corp), including the preparedness for producing the alluvial diagrams and (2) created pages of HTML with Google Maps.

The CC *t* value was denoted by the formula (= CC $\times \sqrt{\frac{n-2}{1-CC\times CC}})$. The significance level was set at Type I error (0.05). A simple regression analysis was performed using MedCalc statistical software, version 9.5.0.0 (MedCalc, New York, NY), to produce a prediction equation. The significance level was also set at Type I error (0.05).

The scatter plot was used to display the relationship between {article citations, MeSH weights} and citations yielded from WOS and PubMed in T100ADHD. All relevant information on the entities can be linked to dashboards on Google Maps. The dashboard of Google Maps is uniquely created using the traditional BibExce software [50]. The guideline using the MSExcel module to draw the alluvial diagram is deposited in Additional file 1.

## Results

### Descriptive statistics (DS)

The T100PEIDs are listed at the link [39]. Readers will be invited to examine all 100 articles included in the study. The number of citations per article ranged from 12 to 123 (average, 37.42) in PubMed and from 8 to 173 (average, 41.26) in WOS on April 12, 2022. No difference in citations was found between WOS and PubMed (*t* = 1.342, df = 198, *p* = 0.181).

The top three countries (the US, China, and South Korea) occupied 81% of T100PEID. The overall T100PEID impact factor (IF=citations/100) is 41.3 (Table 1). Most articles were published in *Spine (Phila Pa 1976)* (23%; IF=41.3), followed by *J Neurosurg Spine* (16%). IF=42.6) and *Eur Spine J (*13%). IF=39.6) (Table 2).

**Table 1:** Distribution of 100 top-cited articles for countries over years (*n* = 100)

Country	2011	2012	2013	2014	2015	2016	2017	2018	n	ci	IF
US	10	3	14	9	8	4	2	4	54	2360	43.7
China	2	3	4		2	3			14	560	40.0
South Korea	2	2	4	2	1	2			13	549	42.2
Netherlands	2		1	1					4	137	34.3
Taiwan	1	1	1						3	104	34.7
Japan			1					1	2	65	32.5
Austria	1								1	26	26.0
Canada		1							1	27	27.0
Denmark			1						1	32	32.0
Germany		1							1	42	42.0
India			1						1	48	48.0
Norway			1						1	37	37.0
Spain	1								1	42	42.0
Sweden					1				1	36	36.0
Turkey	1								1	21	21.0
UK					1				1	40	40.0
n	20	11	28	12	13	9	2	5	100	4126	41.3

*Ci* citations, *IF* impact factor = ci/n

**Table 2:** Distribution of cited articles in journals over the years

Journal	2011	2012	2013	2014	2015	2016	2017	2018	n	ci	IF
Spine (Phila Pa 1976)	192	136	465	67	58			33	23	951	41.3
J Neurosurg Spine	143	73	228	77	54	73		33	16	681	42.6
Eur Spine J	144	96	213		29	33			13	515	39.6
Spine J	64		30	243	119			27	11	483	43.9
J Bone Joint Surg Am	115		43		48		36	14	6	256	42.7
Neurosurgery	76	23			52				4	151	37.8
Neurosurg Focus					32	81			3	113	37.7
World Neurosurg				73		38			3	111	37.0
Cochrane Database Syst Rev				92					2	92	46.0
Expert Rev Med Devices		56		61					2	117	58.5
J Spinal Disord Tech					87				2	87	43.5
Pain Physician			48			38			2	86	43.0
PLoS One		27				30			2	57	28.5
Acta Orthop			37						1	37	37.0
Arch Orthop Trauma Surg					32				1	32	32.0
BMC Musculoskelet Disord		42							1	42	42.0
Clin Neurol Neurosurg			39						1	39	39.0
Clin Orthop Relat Res					61				1	61	61.0
Int J Surg						49			1	49	49.0
Int Orthop			36						1	36	36.0
J Am Acad Orthop Surg			31						1	31	31.0
J Clin Neurosci							36		1	36	36.0
J Orthop Sci								42	1	42	42.0
Singapore Med J	21								1	21	21.0
n	755	453	1170	613	572	342	72	149	100	4126	100

*ci* citations, *IF* impact factor = ci/n

### Major topics or article types with research domains (RD)

**Fig. 1:**
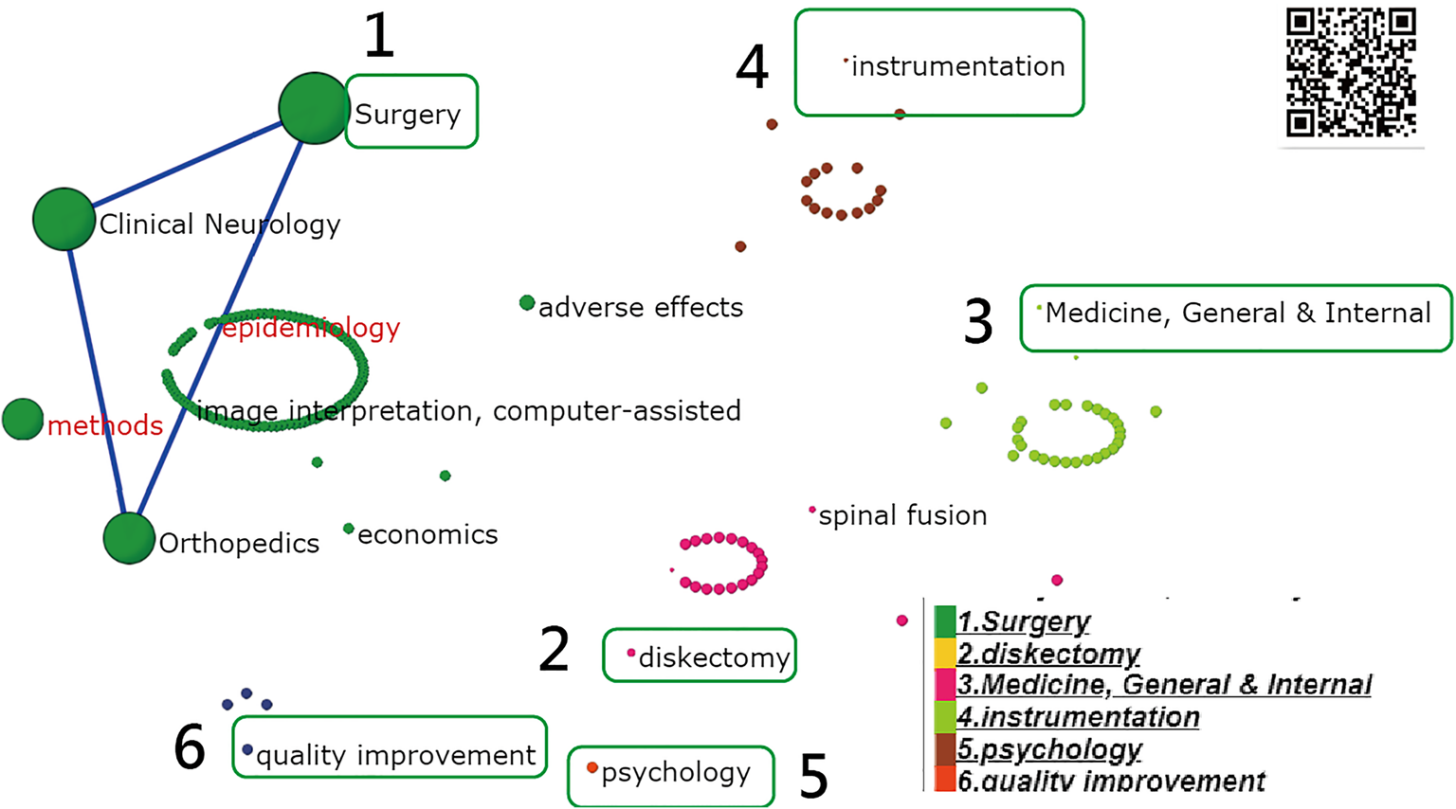
Classification of T100ED on endoscopic discectomy and endoscopic discectomy using social network analysis. Six subject categories in T100PEID classified by SNA (the top three are linked by three blue lines)

Six subject categories were classified using the SNA, including 1. Surgery (57%), 2. diskectomy (12%), 3. Medicine, General & Internal (13%), 4. Instrumentation (11%), 5. Psychology (4%), and 6. Quality improvement (3%) (Fig. 1). The most frequently occurring MeSH term was surgery. No difference was found in article citations among subject categories (*F *= 0.813, *p *= 0.543).

**Fig. 2:**
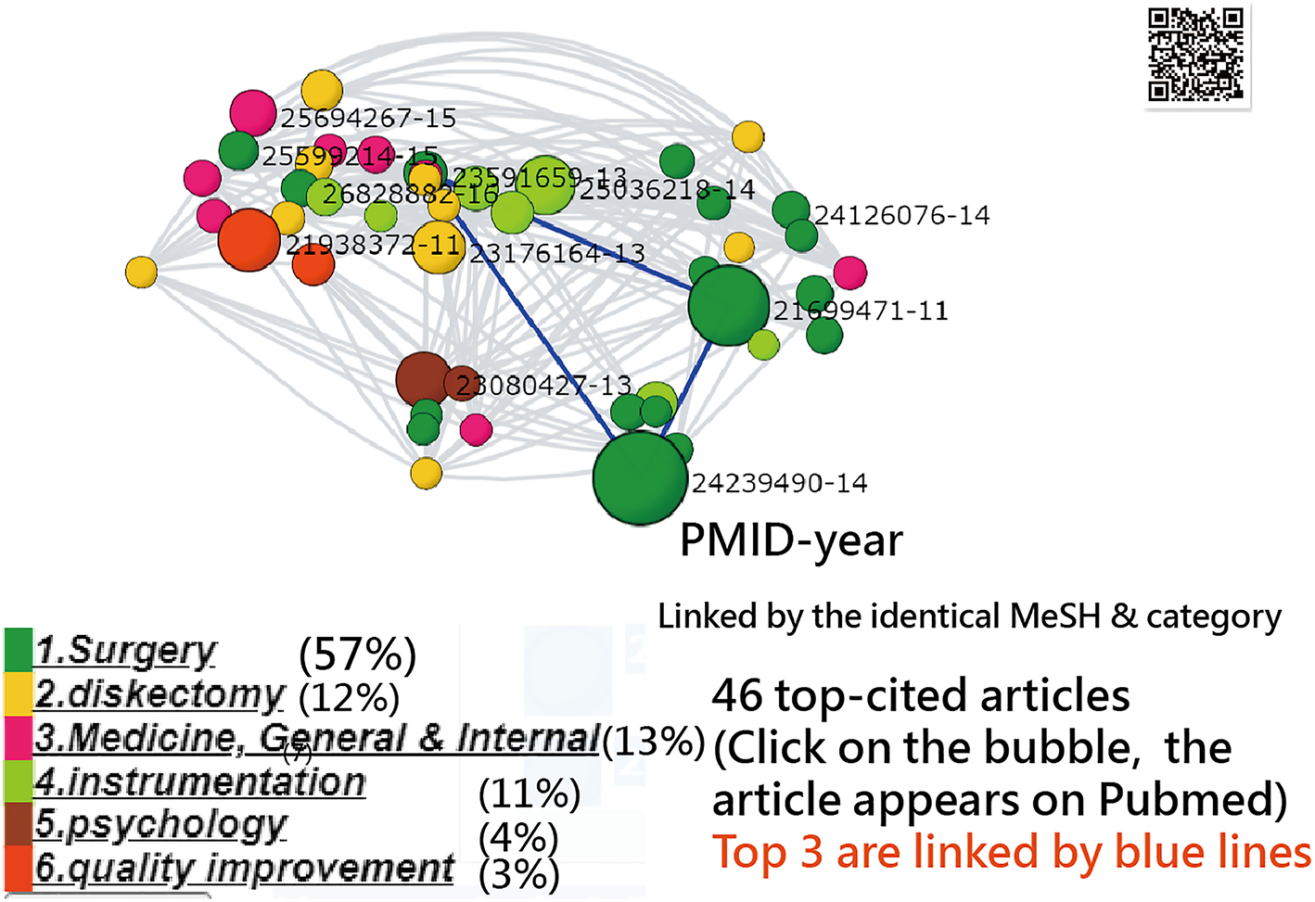
Force-directed network diagram depicts the associations between the articles according to subject categories

The force-directed network diagram depicts the associations between the articles according to the subject categories shown in Figure 2. Articles with more than 39 citations are displayed; only articles sharing more than three identical terms are linked together. The node size and link width are proportional to the number of article citations and shared with similar categories in colors, respectively [17].

### Research achievements (RA) in entities

**Fig. 3:**
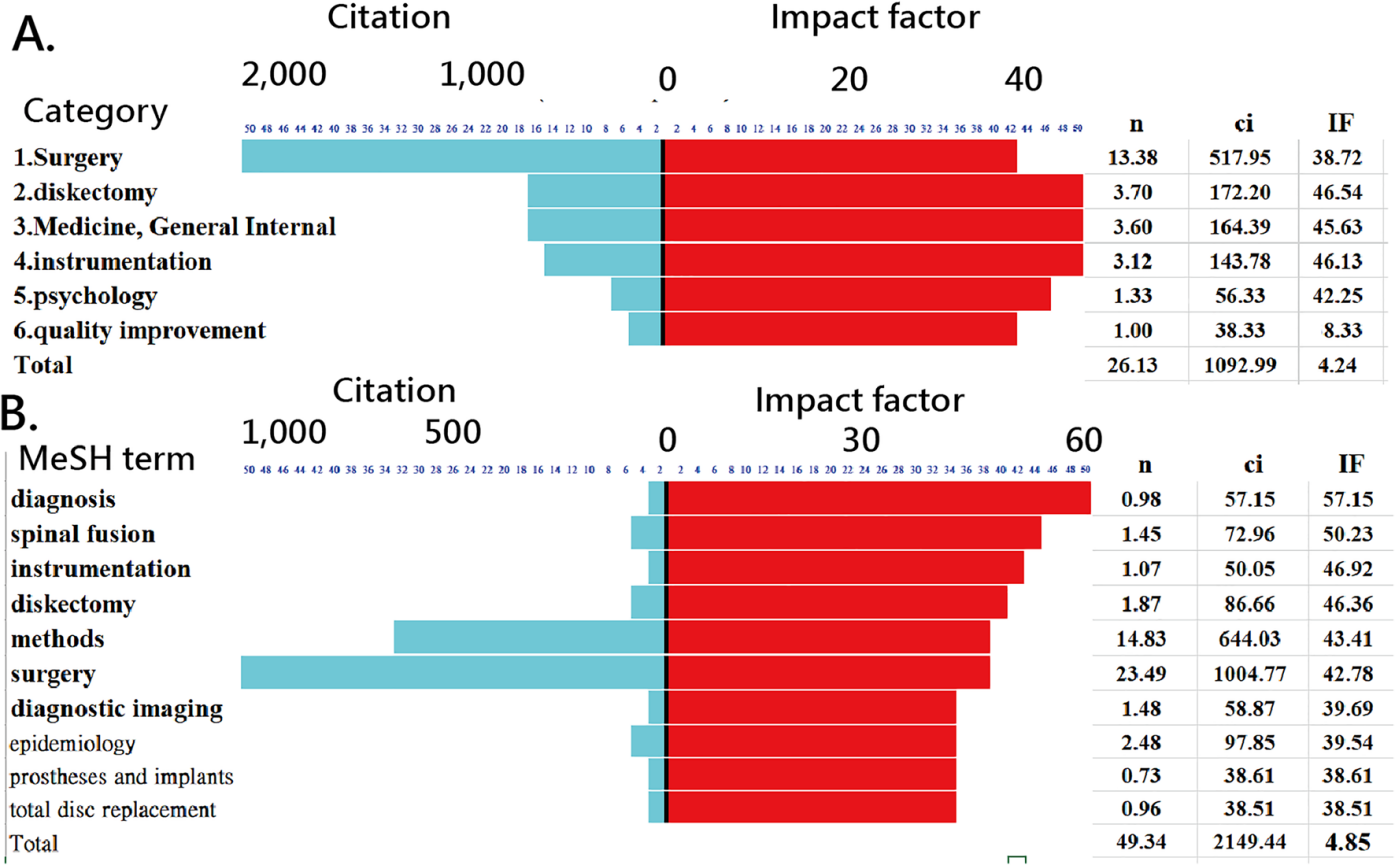
The total number of article citations by subject category and mean citation number after normalization to article counts. Figure 4 100 top-cited articles shown on the impact beam spot (click on the dot to link the article on PubMed; red dots indicate clinical research; the most cited article (PMID: 21699471 published in 2011) [51]

The total number of article citations by subject category and mean citation number (denoted by Ifs) are shown in Fig. 3. We can see that the most cited subject category was surgery, followed by diskectomy and medicine and general internal in panel A of Fig. 3. The weighted number of article citations by major topic MeSH and mean citation number (IFs) yielded in SNA [17] are shown in panel B of Fig. 3. The most cited term is the diagnosis, followed by spinal fusion and instrumentation.

**Fig. 4:**
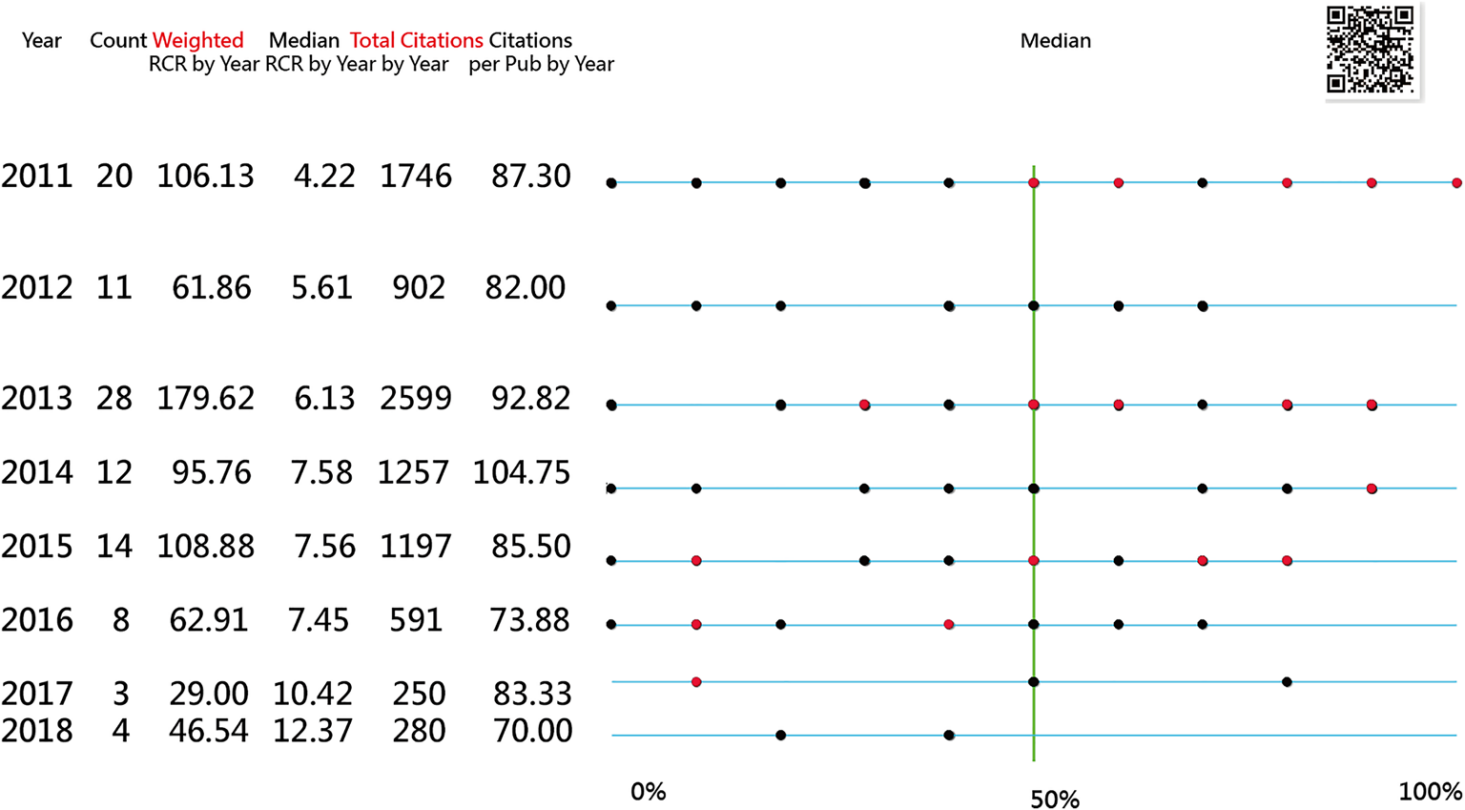
100 top-cited articles shown on the impact beam spot (click on the dot to link the article on PubMed; red dots indicate clinical research; the most cited article (PMID: 21699471 published in 2011) [51]

The T100PEIDs with dots are shown in IBPs (Fig. 4). The red dots represent the articles with the feature of clinical research. The vertical line represents the mean score. *h* = 63 and hT = 54.21 are computed based on WOS. In contrast, *h* = 87 and hT = 58.59 are based on PubMed, indicating that the research achievements (RAs) are slightly higher in the PubMed database.

Readers are invited to scan QR codes on the IBP and click on the dot (e.g., the most left one in 2011). The most cited article authored by D Scott Kreiner from Ahwatukee Sports and Spine in the US state of Phoenix had 123 citations in PubMed (PMID: 24,239,490) [51].

### Visualizations using the alluvial diagram

**Fig. 5:**
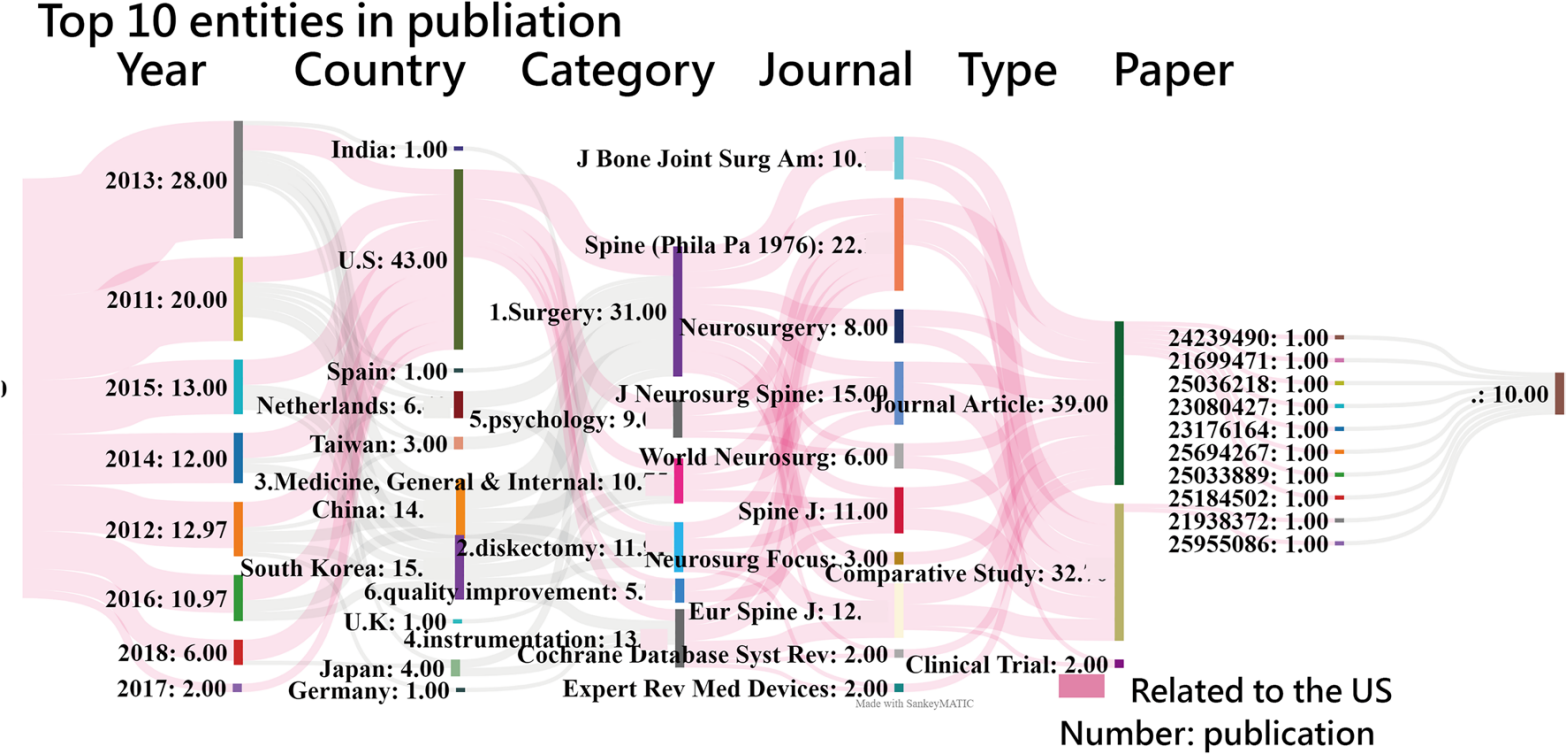
Sankey diagrams depicting the association between article characteristics, including publication year, country of origin, article category, document type, and journal. More to less frequent characteristics are organized from top to bottom. (**i**) The links between characteristics represent the number of shared articles; only those with more than one article are shown, and only those with more than 39 article citations are displayed (density for the US = 0.73)

**Fig. 6:**
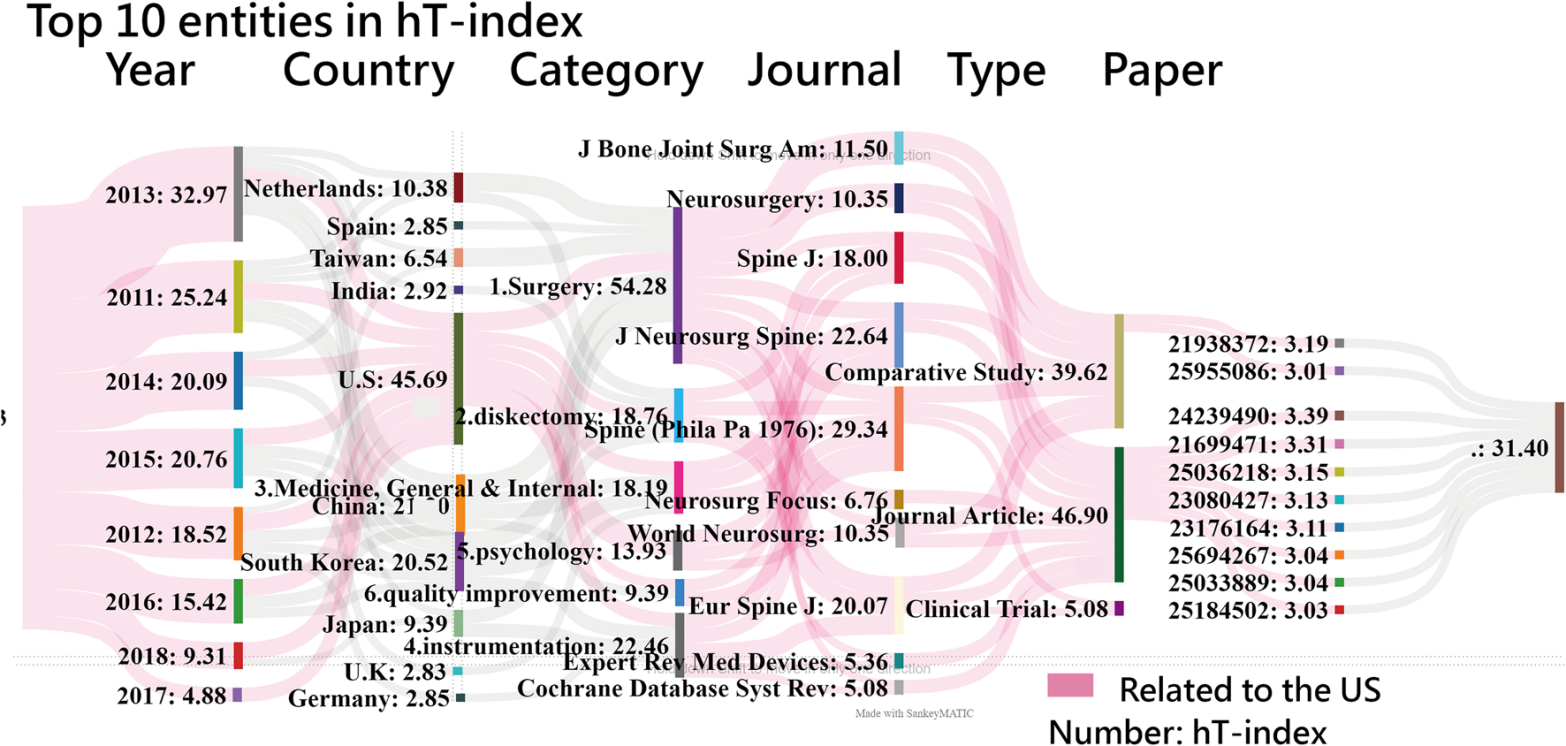
Top 10 entities using the alluvial diagram to display (density for the US = 0.86)

One look is worth a thousand words and quite a few numbers [48]. The top ten entities with higher hT indices are shown on the alluvial diagrams. The red flow means the association with the US with densities of 0.73 and 0.86, respectively, in Figs. 5 and 6. More publications are organized with areas in blocks from higher to lower (Fig. 5). Note that the links between entities represent the number of shared articles; only the top ten entities are displayed.

Similarly, the citation-based alluvial diagram is shown in Fig. 6. The hT indices are numbered and matched to the block height and the flow width. The red-colored flows were mainly designed to represent the entities directly associated with the US.

### Inferring statistics using MeSH terms to predict article citations

**Fig. 7:**
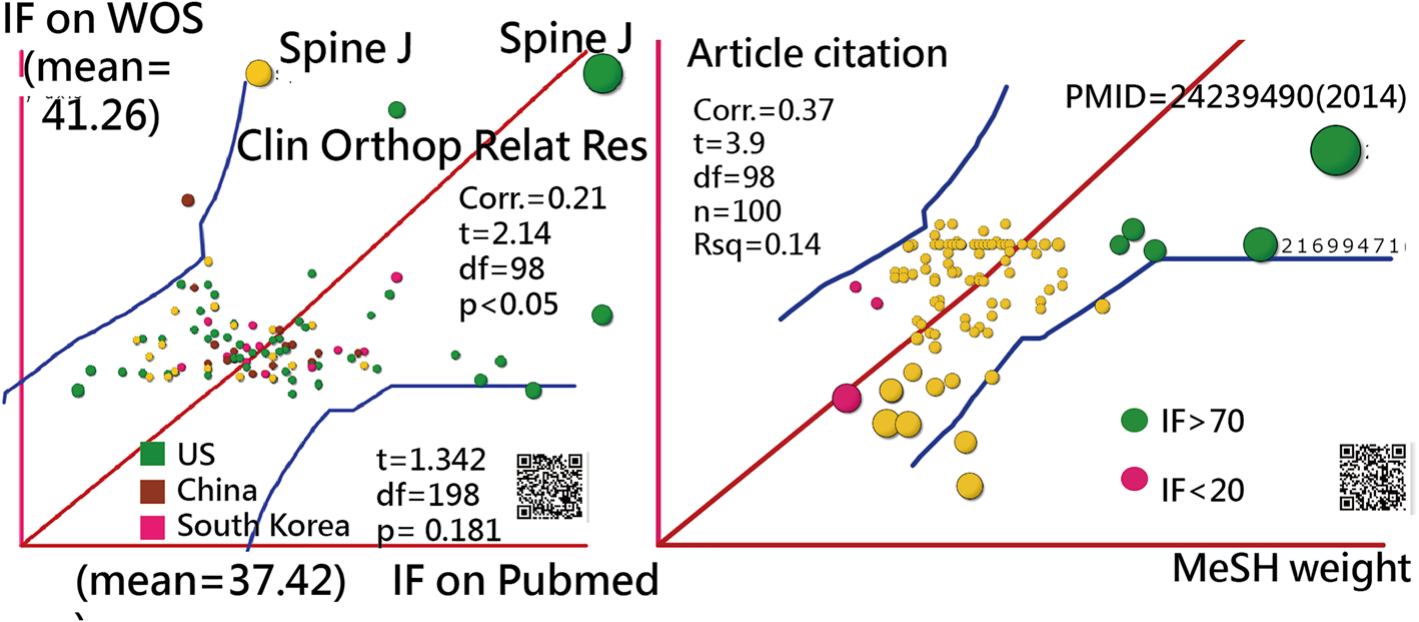
Weighted number of citations based on MeSH terms correlated significantly with the number of article citations (Pearson’s *r* = 0.37; *p* < 0.001)

No difference was found in citations from either WOS or PubMed (*t*=1.342, df=198, *p*=0.181) based on the independence *t* test. However, the correlation coefficient is significant at 0.21 (*t*=2.14, df=98, *p*<0.05). We can see that the highest number of citations was from the US in green and the journal *Spine J* at the top-right corner in the left panel of Figure 7.

MeSH terms were evident in the prediction power of the number of article citations (CC = 0.37, *F* = 15.21, df = 98, *p* < 0 0.001). The prediction equation is article citation = 13.7548 + 0.7344 $\times$ MeSH weight.

### Online dashboards shown on Google maps

All dashboards in the figures immediately appear once the QR code is clicked. Readers are advised to examine the details of each entity's information on dashboards.

## Discussion

There were the following entities with the largest number of contributions (denoted by hTs) made to PEID in T100PEID: 2013 (32.97) in years, the US (45.69) in countries, 1.surgery(54.28) in subject categories, *Spine(Phila Pa 1976)*(29.34) in journals, Journal Article(46.9) in document types, and PMID=24239490(3.39) in articles. A proverb goes that one look is worth a thousand words and quite a few [48]. In Figure 6, we present the top ten entities with the highest hTs, and we were able to achieve our goal of displaying influential entities in T100PEID on the alluvial diagram. Traditionally, more than six tables or figures are required to display influential entities with contributions to the scholarly field (or discipline, e.g., PEID). The second research goal was also achieved to evidence MeSH terms in the prediction power of the number of article citations (*F*  = 15.21; *p*  < 0 .001).

### Additional information

T100PEIDs from PubMed's database were categorized into six categories based on their characteristics. We used alluvial diagrams and network analysis to determine the features and underlying relationships in T100PEIDs. Using these concise diagrams, spine surgeons may find relevant articles more efficiently, facilitating evidence-based decision-making for patients with PEID.

SNA was used to determine the article subject categories associated with frequent citations. A combination of both publications and citations contributed to the highest hT index for the subject category “surgery”. There may be a reason for this result because spine surgeons have to realize that PEID-related articles with higher hTs are present in the domain of surgery research. Of the common major topic MeSHs listed in these top-cited articles, “diagnosis” had the highest IFs. This may be partly attributed to the critical role of diagnosis revealed in T100PEIDs. This article applied SNA to describe the classification of PEID-related articles. With these classifications for PEID, spine surgeons may use the classification as an international communication tool to discuss any topic regarding PEID. From our point of view, the classification approach can be applied to other scientific studies and not limited to PEID.

The alluvial diagram has been applied to bibliometric analysis in two studies [21, 52]. The alluvial is appropriate for their graphs due to categorical dimensions instead of steps (or years) on the x-axis, referring to the definitions of Sankey diagrams [38].

Someone pointed out that while Sankey diagrams are better known, alluvial plots are generally a good deal easier to generate [53]. It is only valid because the data are simple enough as the software [54] to draw the alluvial without taking the weights (e.g., citations and hT-indices) into account. As such, it is harder to draw the alluvial than the Sankey, particularly in three situations: (1) the weights are yielded by SNA and proportionally allocated to nodes and arcs; (2) the flows between dimensions are backward extracted from the SNA [31] instead of forward to the step-by-step process in the Sankey; and (3) Sankey diagrams placing nodes more freely than on alluvial plot that instead requires their nodes to be aligned and cannot be randomly placed [38]. We have not seen software to take those situations into account for drawing the alluvial as we provided the teaching material in Additional file 2.

Additionally, the reasons for demonstrating the hT-index in this study are because (1) the hT-index has an identical h-core with the h-index [15], (2) there is a strong association with the h-index, and (3) all publications and citations are taken into account to overcome the disadvantage associated with many bibliometric indices.

To date, no studies related to PEID have been identified in PubMed. The current study on T100PEID is the first to use bibliometric analysis in the PEID field. In Fig. 4, a dashboard-type IBP provides information rather than the 100 articles listed across all papers in a study. This is a unique and modern approach never seen before in the literature. The IBP presents the T100PEID in a single view and provides more context than a single metric, such as a citation metric (or the h-index [15]) in bibliometrics. Bibliometric analysis can be advanced in this manner.

### Three most cited articles

The most cited article in T100PEID was written by Kreiner et al. This study was published in Spine J [51] in 2014 and categorized as “Medicine, General & Internal”. In this article, the authors summarize (1) the techniques used by evidence-based medicine and provide the best available evidence to assist practitioners in the care of patients with symptomatic lumbar disc herniation with radiculopathy and (2) the complete guideline document for future research.

The second most highly cited article was written by Coric et al. and published in 2011 in J Neurosurg Spine [55], which was classified as “surgery” in our study. This was a prospective, randomized US FDA Investigational Device Exemption (IDE) pivotal trial conducted at 21 centers across the US, finding that KineflexC (SpinalMotion, Inc.) was associated with a significantly higher success rate than fusions while maintaining motion at the index level. Even though there were significantly fewer Kineflex C patients with severe adjacent-level radiographic changes following the 2 year follow-up, these results indicate that Kineflex C CTDR is a viable alternative to anterior cervical discectomy and fusion (ACDF) in select patients with cervical radiculopathy.

The third most cited article appeared in J Bone Joint Surg Am by Sasso et al. in 2011 [56] and was classified as “surgery” in our study. This article addresses that the arthroplasty cohort continued to show significantly greater improvements in the Neck Disability Index, neck pain score, arm pain score, and Short Form-36 physical component score, as well as the primary outcome measure, overall success, at 48 months following surgery.

Thus, spine surgeons should pay special attention to PELD, LDH, PELD, PETD, and PEID [1–4].

### Implications and changes

This study has several noteworthy features. In the first instance, the hT-index with decimal places can enhance the original h-index in terms of identifying the research accomplishments and rankings of a given group [57]. To measure the achievements of researchers and research institutions, we proposed using the hT index.

The second feature is that alluvial was used to highlight a few vital entities and proved to be viable and feasible in bibliometrics.

The third feature is the use of IBPs [43, 44], providing authors with a brand-new representation of every academic article, particularly with research achievements denoted by the hT-index instead of the median percentile only shown to authors of core articles in Web of Science (WOS) [58, 59].

We presented those entities with the highest hTs in the alluvial diagram. As a consequence, more than six tables or figures are required to demonstrate the important entities that have contributed to the scholarly field (or discipline, e.g., PEID).

In addition, the classification of subject categories using SNA is objective and unique when compared to previous studies using manual methods [21] or document types determined by PubMed [52]. Despite the fact that no difference was found in the citations between the subject categories (*F* = 0.813, *p* = 0.543), the evidence suggests that the classification method is valid and worth recommending to future researchers. Although the hT-index is more complex to compute than the h-index, the problem can be solved by a dedicated software program. The hT-index computation has been analyzed at the link [60], which provides readers with the programming codes for understanding how the hT-index is calculated within a second.

### Limitations and suggestions

Further research should examine a number of issues. The first concern is that the software used to draw the alluvial diagrams [37, 38] is not unique and irreplaceable. Several other software packages [54, 61, 62] make it easy to draw the alluvial (or Sankey) online. However, they do not meet the three requirements (i.e., weights derived from the arcs in SNA, flows between dimensions backward derived from the SNA, and nodes aligned and vertically aligned to the respective dimension on the x-axis) required in this study.

Second, dashboards in this study are displayed on Google Maps. These installments are not free of charge because Google Maps requires a paid project key for using the cloud platform. Therefore, it is difficult for other authors to replicate the usage in a short period of time.

Third, the hT-index calculated by adding up the weights in the Ferrers tableau (i.e., all the cited papers in the list) requires considerable computation. As a result of the improved hardware, the time-consuming task is now trivial and equivalent to the computation of other bibliometric indices using dedicated software.

Fourth, although the IBP in this study was produced online [47], the research achievements are determined by many other factors (e.g., the journal impact factor, JIF) that should be considered when drawing the IBP (e.g., using the JIF-based hT index to draw the IBP).

Fifth, only a few dimensions were selected in the alluvial diagram. Other important categories (e.g., research institutes and influential authors in T100PEID) are required to display on the alluvial diagram simultaneously. Future studies are recommended to involve more dimensions on the x-axis on the alluvial diagram.

Finally, although T100PEIDs were extracted mainly from PubMed, the results were different in articles retrieved from other databases (e.g., Google Scholar, Scopus, and WOS). Future studies are required to extract T100PEID from more bibliometric databases.

## Conclusion

By drawing the network characteristics in T100PEID, a breakthrough was achieved. MeSH terms may be used to classify article subject categories and predict T100PEID citations. In future studies, the alluvial diagram can quantify bibliometric data on 100 top-cited articles rather than focusing on PEID, as in this study.

## Supplementary Information

**Additional file 1.** Dataset and module used in this study.

**Additional file 2.** Examples illustrated for drawing the Alluvial diagram.

## Data Availability

All data used in this study are available in Supplemental Digital Content.

## Abbreviations

CC: Correlation coefficient
IF: Impact factor
IMP: Impact beam plot
MeSH: Medical subject heading
PEID: Percutaneous endoscopic interlaminar discectomy
RA: Research achievement
RD: Research domain
SNA: Social network analysis
WOS: Web of Science
