# Supplementary material for: Citation analysis of the 100 top-cited articles on discectomy via endoscopy since 2011 using alluvial diagrams: bibliometric analysis
Source: Eur J Med Res. 2022 Sep 1;27:169. doi: 10.1186/s40001-022-00782-0 (PMC9438267; doi:10.1186/s40001-022-00782-0)
Supplement: Supplementary file 2 — Additional file 2. Examples illustrated for drawing the Alluvial diagram. [file 40001_2022_782_MOESM2_ESM.pdf]

Step1: Extract data from Pubmed

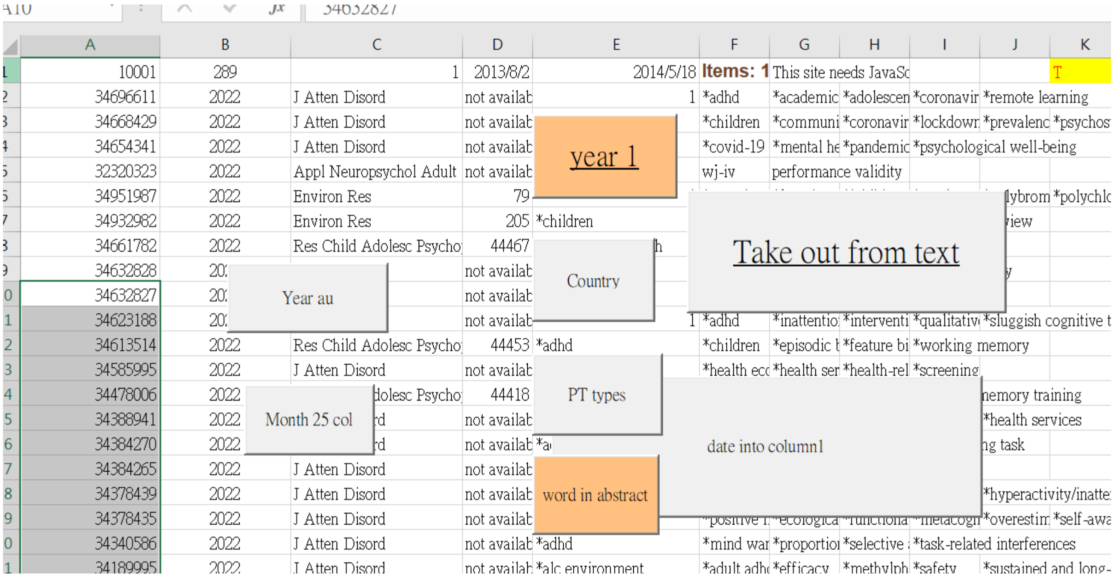

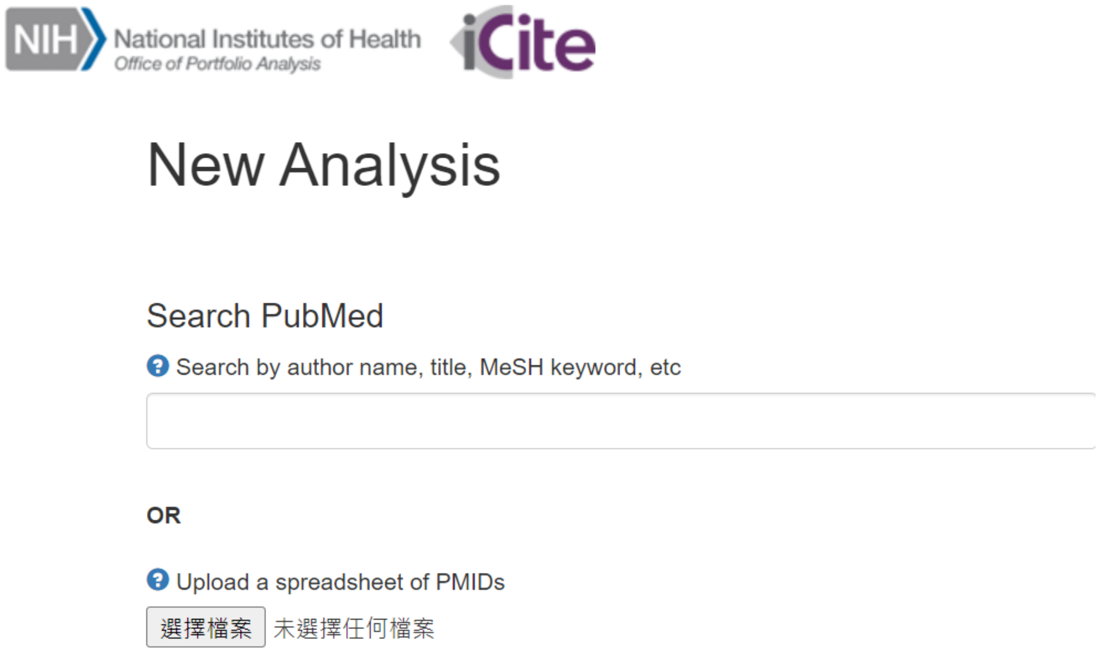

Step2: Take out the top 10 with higher total citations(takeoutmeshcountintop10)

	A	B	C	D	E	F	[illegible]
1	RCT	14644	#VALUE!	journal	Type	country	state
2	6.05	28006890	2021	Minerva Pediatr (Torino)	Journal Article	Italy	Italy
3	0	29294948	2021	J Interpers Violence	Journal Article	U.S	Flori
4	0	29233062	2021	J Atten Disord	Journal Article	Israel	Israe
5	4.3	29284437	2017	BMC Pediatr	CommandButton1	ael	Israe
6	0.24	29281965	2017	J Neurodev Disord	[illegible]	S	Tenr
7	3.23	29267405	2017	PLoS One	[illegible]	nland	Finla
8	2.45	29246205	2017	BMC Psychiatry	Journal Article	Sweden	Swed
9	0	29292981	2017	Lakartidningen	Letter		
10	0.13	29188613	2017	Zhonghua Yi Xue Yi Chu	Journal Article	China	Sich
11	0	29262484	2017	Zhonghua Yu Fang Yi Xu	Journal Article	China	Anhu
12	1.05	28753050	2017	Subst Use Misuse	Journal Article	U.S	Ohic
13	1.07	29280392	2017	J Behav Addict	Journal Article	Germany	Gerr
14	1.76	[illegible]	2017	Psychol Assess	Journal Article		

Step3: Compute the hT-index for the top three in each article category

	A	B	C	D	E	F	G	H	I	J
1	914.17	WGS	Recent stag	WES	Early stage	4	4	3.5	4	
2	ALL	47.02		23	71.32	69.82	67.14	47.13	46.89	[illegible]
3	Early	37.55		18	71.32	47.13	46.89	44.43	40.31	[illegible]
4	Recent	36.26		17	69.82	67.14	42.42	35.11	33.89	[illegible]
5	2020	23.68	35.83	12	33.89	26.27	25.37	21.9	21.45	[illegible]
6	2014	20.1	0	9	[illegible]	[illegible]	24.51	14.24	13.86	[illegible]
7	2019	21.21	31.67	10	Taking out from [illegible]	[illegible]	29.12	18.19	17.34	[illegible]
8	2018	19.88	31.28	10	[illegible]	[illegible]	26.34	16.84	15.73	[illegible]
9	2012	21.06	23.71	11	44.43	21.89	20.89	16	14.86	[illegible]
10	2016	19.59	21.38	9	[illegible]	25.83	23.37	15.5	14.36	[illegible]
11	2013	20.83	18.57	10	Sort	28.62	24.16	18.06	15.18	[illegible]
12	2015	18.65	18.43	8	[illegible]	30.95	19.11	17.11	12.94	[illegible]
13	2017	17.47	17.67	8	21.54	19.35	18.47	11.5	10.39	
14	2011	18.98	17.61	9	hT	18.9	13.8	12.65	11.67	[illegible]
15	J Atten Disord	19.66	16.91	10	[illegible]	16.16	14.09	13.87	12	[illegible]
16	J Child Psychol Psychiatry	15.76	16.12	8	[illegible]	21.44	15.18	14.98	11.5	[illegible]
17	J Am Acad Child Adolesc Psych	15.92	16.14	8	36.39	14.36	12.94	10.88	10.73	[illegible]
18	[illegible]	[illegible]	[illegible]	[illegible]	[illegible]	[illegible]	[illegible]	[illegible]	[illegible]	[illegible]

Step4: Take out the citations over the years for top 3 in citation of entities

Enity		hT
ALL		47.02
Early		37.55
Recent		36.26
	2020	23.68
	2014	20.1
	2019	21.21
J Atten Disord		19.66
J Child Psychol Psychiatry		15.76
J Am Acad Child Adolesc Psychiatry		15.92

Journal Article	41.37
Comparative Study	16.25
Clinical Trial	6.38
U.S	33.73
U.K	24.1
Netherlands	17.15
Germany	14.28
New York	18.67
Canada	14.58
King's College London(UK)	13.59
Karolinska Institutet(Sweden)	11.9
University of California(California)	8.97
Faraone, Stephen V(New York)	9.49
Becker, Stephen P(Ohio)	9.07
Cortese, Samuele(U.K)	8.97
attention deficit disorder with hyperactivity	11.05
mindfulness	3.23
conduct disorder	3.16

Step5: obtain the trend data (e.g., citations or RCT) for each target element of entity

	A	B	C	D	E	F	G	H	I	J	K	L
1	for hT	2011	2012	2013	2014	2015	2016	2017	2018	2019	2020	2021
2	ALL(count)	1069	1127	1232	1342	1318	1297	1310	1314	1341	1471	1584
3	ALL(citation)	1520.54	1723.61	1707	1850.7	1636.21	1717.55	1601.02	1742.98	1849.84	2176.78	805.18
4	2012											
5	J Atten Disord	77.28	84.56	94.51	67.67	110.3	119.52	149.75	181.86	224.38	520.31	165.19
6	J Child Psychol Psychiatry	42.51	47.58	74[illegible]	[illegible]	[illegible].43	56.1	49.97	69.55	60.95	57.28	40.29
7	J Am Acad Child Adolesc Psychiatry	69.13	52.81	39[illegible]	[illegible]	[illegible].14	65.79	41.56	26.53	35.89	31.08	59.69
8	Journal Article	1281.51	1533.74	1471.8	1690.52	1451.33	1565.36	1479.39	1536.85	1752.31	1958.71	797.29
9	Comparative Study	156.17	129.73	140.52	71.34	97.56	83.5	58.06	92.5	34.27	57.7	2.64
10	Clinical Trial	35.1	16.45	28.27	11.36	24.61	13	15.5	10.7	12.23	18.09	0
11	U.S	639.01	828.2	628.64	590.76	539.79	602.56	476.07	580.02	548.98	729.46	347.9
12	U.K	342.4	307.54	413.54	404.42	291.58	416.62	261.12	377.28	231.32	378.5	121.06

Step6: using the Sankey to draw the trend for each entity(sankeysimple)

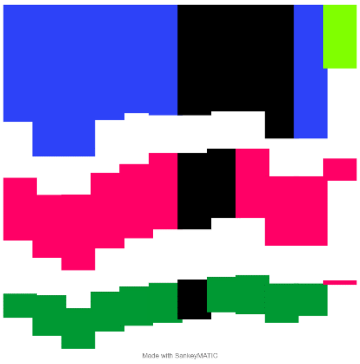

Step 7: using the Alluvial to draw the feature of top three in hT-index(datasetsankey)

(1) Relations between entities using SNA based coword relationship.

Note. Only the entities required are put into the SNA to save time running the SNA.

(2) Transform the hTs into the relations and run the Sankey diagram

(3) Download the image to make the data presentations.

Step 8 Alluvial diagram

	J	K	L	M	N	O	P	Q	R	
1	Journal	ddd	Type	ddd	Category	ddd	Country	ddd	PMID	RCI
2	Spine (Phila P.	29.36	Journal Article	47.69	1.Surgery	45.99	U.S	47.31	24239490	242
3	J Neurosurg Sp	22.66	Comparative Study	39.77	3.Medicine	20.5	China	21.22	21699471	216
4	Eur Spine J	20.08	Clinical Trial	5.07	2.diskector	19.64	South Korea	20.51	21938372	219
5	Spine J	18.01			4.instrume	18.64	Netherlands	8.25	25036218	250
6	J Bone Joint S	11.5			5.psycholo	8.7	Taiwan	6.54	23080427	230
7	Neurosurgery	8.36			6.quality in	6.81	Japan	4.73	23176164	231
8	Neurosurg Foc	6.76					India	2.92	25694267	256
9	World Neurosu	6.76					Germany	2.85	25033889	250
10	Expert Rev Me	5.36					Spain	2.85	25184502	251
11	Cochrane Data	5.08					U.K	2.83	25955086	259
12										
13										
14										

	A	B	C	D	E	F	G	H	I	J	K	L
1	Buffer	Year	0.73		#ff0066	U.S					Year	ddd
2	0	2011	U.S	5.91	#ff0066							146.79
3	1.Surgery	2011	China	4.24								
4	3.Medicine, Genera	2011	South Korea	3.42								
5	2.diskectomy	2011	Netherlands	2.75								
6	4.instrumentation	2011	Taiwan	2.18								
7	5.psychology	2011	Spain	2.85		21.35						
8	6.quality improvem	2012	U.S	5.91	#ff0066							
9	Spine (Phila Pa 19	2012	China	4.24		31.5						
10	J Neurosurg Spine	2012	South Korea	3.42								
11	Eur Spine J	2012	Taiwan	2.18								
12	Spine J	2012	Germany	2.85								
13	J Bone Joint Surg	2013	U.S	5.91	#ff0066							
14	Neurosurgery	2013	China	4.24								
15	Neurosurg Focus	2013	South Korea	3.42								
16	World Neurosurg	2013	Netherlands	2.75								
17	Cochrane Database	2013	Taiwan	2.18								

To count

Arc color

To citation

Node links compared

To dd2 html

Weights

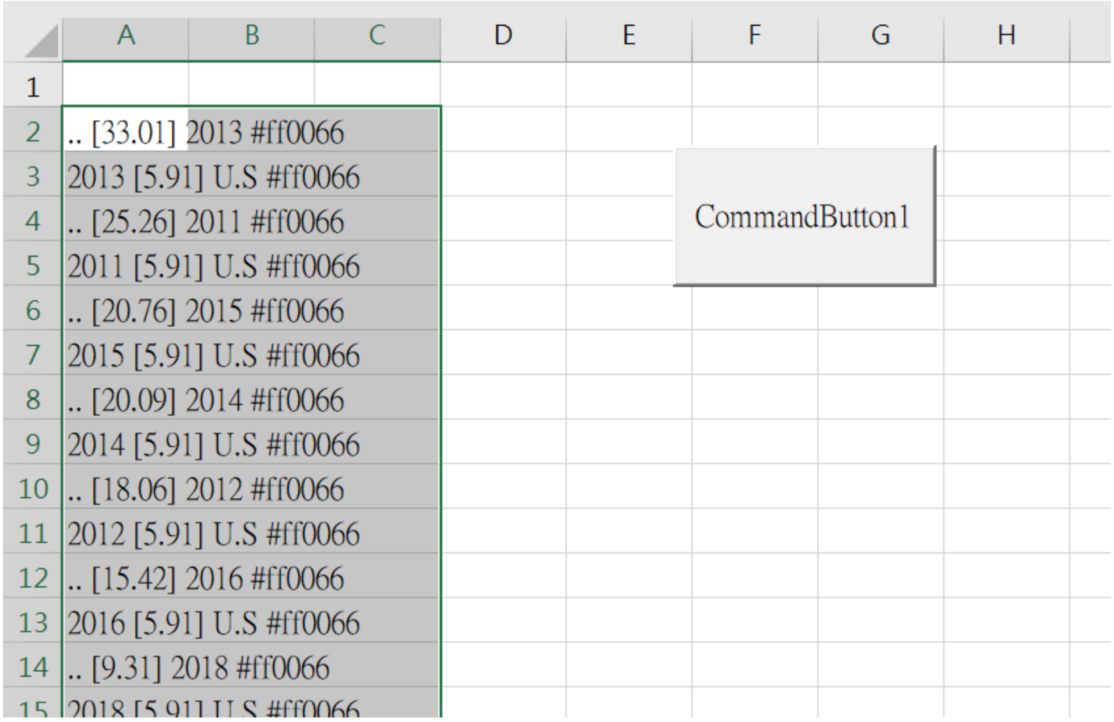

A B C D E F G H
.. [33.01] 2013 #ff0066
2013 [5.91] U.S #ff0066
.. [25.26] 2011 #ff0066
2011 [5.91] U.S #ff0066
.. [20.76] 2015 #ff0066
2015 [5.91] U.S #ff0066
.. [20.09] 2014 #ff0066
2014 [5.91] U.S #ff0066
.. [18.06] 2012 #ff0066
2012 [5.91] U.S #ff0066
.. [15.42] 2016 #ff0066
2016 [5.91] U.S #ff0066
.. [9.31] 2018 #ff0066
CommandButton1

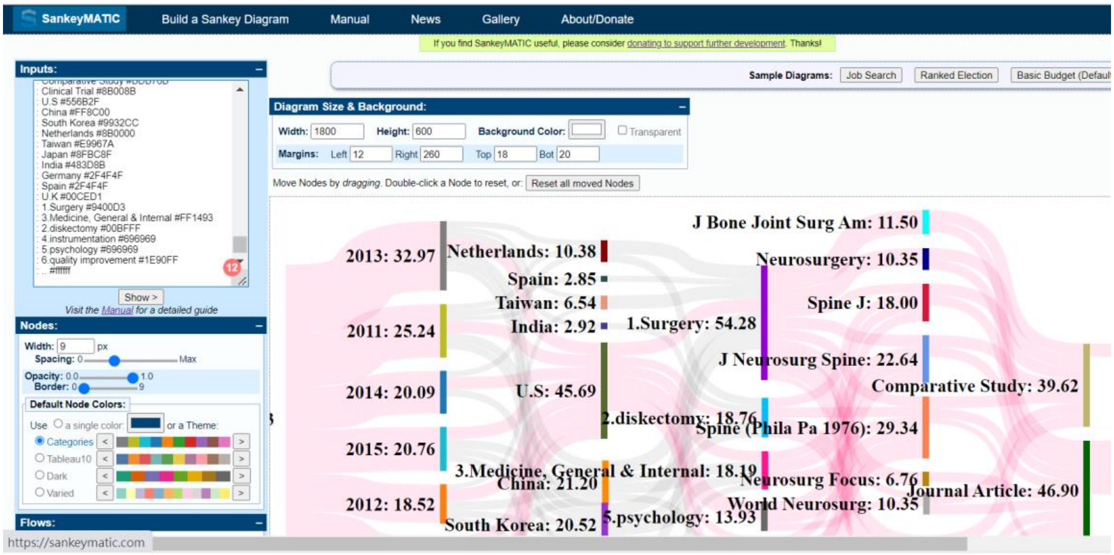

SankeyMATIC
Build a Sankey Diagram
Manual
News
Gallery
About/Donate
Inputs:
Nodes:
Flows:
Diagram Size & Background:
2013: 32.97
2011: 25.24
2014: 20.09
2015: 20.76
2012: 18.52
Netherlands: 10.38
Spain: 2.85
Taiwan: 6.54
India: 2.92
U.S: 45.69
China: 21.20
South Korea: 20.52
1.Surgery: 54.28
2.diskectomy: 18.76
3.Medicine, General & Internal: 18.19
5.psychology: 13.93
J Bone Joint Surg Am: 11.50
Neurosurgery: 10.35
Spine J: 18.00
J Neurosurg Spine: 22.64
Spine (Phila Pa 1976): 29.34
Neurosurg Focus: 6.76
World Neurosurg: 10.35
Comparative Study: 39.62
Journal Article: 46.90
https://sankeymatic.com
